# Cardiorespiratory Assessments in Panic Disorder Facilitated by Wearable Devices: A Systematic Review and Brief Comparison of the Wearable Zephyr BioPatch with the Quark-b2 Stationary Testing System

**DOI:** 10.3390/brainsci13030502

**Published:** 2023-03-16

**Authors:** Daniela Caldirola, Silvia Daccò, Massimiliano Grassi, Alessandra Alciati, William M. Sbabo, Domenico De Donatis, Giovanni Martinotti, Domenico De Berardis, Giampaolo Perna

**Affiliations:** 1 Department of Biomedical Sciences, Humanitas University, Via Rita Levi Montalcini 4, 20090 Pieve Emanuele, Italy; 2 Department of Clinical Neurosciences, Villa San Benedetto Menni Hospital, Hermanas Hospitalarias, Via Roma 16, 22032 Albese con Cassano, Italy; 3 Humanitas San Pio X, Personalized Medicine Center for Anxiety and Panic Disorders, Via Francesco Nava 31, 20159 Milan, Italy; 4 Humanitas Clinical and Research Center, IRCCS, Via Manzoni 56, 20089 Rozzano, Italy; 5 Department of Neuroscience, Imaging and Clinical Sciences, University “G. d’Annunzio”, 66100 Chieti, Italy; 6 Department of Mental Health, NHS, ASL 4 Teramo, Contrada Casalena, 64100 Teramo, Italy

**Keywords:** panic disorder, respiration, cardiac function, digital psychiatry, wearable devices, breathing rate, heart rate, Quark-b2, Zephyr BioPatch

## Abstract

Abnormalities in cardiorespiratory measurements have repeatedly been found in patients with panic disorder (PD) during laboratory-based assessments. However, recordings performed outside laboratory settings are required to test the ecological validity of these findings. Wearable devices, such as sensor-imbedded garments, biopatches, and smartwatches, are promising tools for this purpose. We systematically reviewed the evidence for wearables-based cardiorespiratory assessments in PD by searching for publications on the PubMed, PsycINFO, and Embase databases, from inception to 30 July 2022. After the screening of two-hundred and twenty records, eight studies were included. The limited number of available studies and critical aspects related to the uncertain reliability of wearables-based assessments, especially concerning respiration, prevented us from drawing conclusions about the cardiorespiratory function of patients with PD in daily life. We also present preliminary data on a pilot study conducted on volunteers at the Villa San Benedetto Menni Hospital for evaluating the accuracy of heart rate (HR) and breathing rate (BR) measurements by the wearable Zephyr BioPatch compared with the Quark-b2 stationary testing system. Our exploratory results suggested possible BR and HR misestimation by the wearable Zephyr BioPatch compared with the Quark-b2 system. Challenges of wearables-based cardiorespiratory assessment and possible solutions to improve their reliability and optimize their significant potential for the study of PD pathophysiology are presented.

## 1. Introduction

The involvement of respiratory and cardiac functions in panic attacks (PAs) and panic disorder (PD) has been extensively documented. Prominent respiratory symptoms during PAs and respiratory complaints not occurring during PAs during daily life are common in patients with PD [1,2,3,4]. Furthermore, palpitations and accelerated heart rate (HR) are frequent symptoms both during and beyond PA events [1,2,3,4]. Empirical evidence has pointed to respiratory dysregulation as a biomarker of panic vulnerability [5,6] and several abnormalities in resting-state respiratory patterns have repeatedly been documented in patients diagnosed with PD when compared with healthy controls (HCs) or patients with other anxiety disorders [4,7,8,9]. Subclinical abnormalities in autonomic and cardiac function have also been found in patients with PD [4,7,10,11,12]. Respiratory and autonomic instability preceding PA events have been observed [13].

Although these findings are robust and consistent across a plethora of studies, most research on panic pathophysiology has been conducted in the laboratory through invasive recording systems, particularly respiratory assessments, which usually require facemasks or mouthpieces. Even when ambulatory monitoring in a natural environment was conducted, complex and uncomfortable portable devices were used [13,14,15]. This research approach has yielded data with high internal validity and excellent reproducibility. Nevertheless, concerns have been raised about the invasiveness of data capture. The detected cardiorespiratory abnormalities of patients with PD may have resulted from their increased sensitivity to unfamiliar or stressful conditions in the laboratory or invasive instrumentation, rather than being consistent features of their daily lives [16,17]. Consistent with this concern, preliminary results based on a non-invasive ambulatory monitoring system found only limited support for previous laboratory-detected respiratory abnormalities in patients with PD [16,17]. 

Therefore, much more research outside of the laboratory is required to provide deeper insights into cardiorespiratory functioning in patients with PD, which may contribute to diagnostic and therapeutic advances for this disorder [4,18]. Recent technological advances can facilitate this type of research. The integration of miniaturized physiological sensors into “smart” non-invasive wearable devices can provide more accessible and less invasive real-time physiological data collection. Advances in machine-learning techniques can enable patterns to be more easily detected or offer predictive models from large and highly composite data samples [19,20]. 

However, the use of wearable technology devices to record physiological signals in naturalistic environments may also have shortcomings. Measurement artifacts and variability may occur when patients move freely without supervision. Moreover, wearables may be less precise than stationary instruments that are purposefully designed for accurate data capture. Wearables-based respiratory recordings are particularly challenging and present technical problems. Since wearable devices do not directly measure air flow, it is difficult to obtain accurate measurements of factors such as minute ventilation or tidal volume [21,22], or estimates of optimal respiratory waveforms [23,24]. These critical aspects might undermine the detection of subtle, subclinical respiratory abnormalities in patients with PD, especially in dynamic conditions. 

The present manuscript includes two sections and a general discussion. The main section (Part 1) is a systematic review of studies that carried out respiratory and/or cardiac measurements on patients with PD using wearable devices outside of a laboratory setting, that is, in the natural environment. No reviews on this topic were available, except for one published in 2019, which had different aims; these included studies recording electrocardiogram signals with wearable or portable devices on patients with different psychiatric disorders [25]. Thus, we present a critical overview of this topic, evaluate whether advances have been made in recent years, and discuss the advantages and disadvantages of using less invasive wearable devices for cardiorespiratory-data capture. 

Additionally, in Part 2, we present the preliminary results of a pilot study we conducted to evaluate the accuracy of the wearable Zephyr BioPatch in the estimation of heart rate (HR) and breathing rate (BR) using the Quark-b2 stationary testing system as a benchmark. The inclusion of our explorative results is intended to expand the findings we systematically reviewed and offer an adjunctive contribution to the open issue of these wearables’ accuracy in the measurement of cardiorespiratory parameters. 

## 2. Part 1: Systematic Review

### 2.1. Materials and Methods

This review was conducted according to the Preferred Reporting Items for Systematic Reviews and Meta-Analyses (PRISMA) 2020 statement [26]. This protocol was not previously registered. 

#### 2.1.1. Search Strategy 

A database search of peer-reviewed scientific literature written in English was conducted using PubMed, PsycINFO, and Embase, from their respective inception dates to 30 July 2022. 

The following keywords were used in the PubMed search: (Panic[title/abstract] AND (watch[title/abstract] OR wearable*[title/abstract] OR device*[title/abstract] OR ambulatory[title/abstract])) AND (“heart rate” [title/abstract] OR HRV[title/abstract] OR cardiorespir*[title/abstract] OR respir* [title/abstract] OR breath*[title/abstract] OR cardiac[title/abstract] OR pulse[title/abstract] OR “tidal volume” [title/abstract] OR variability[title/abstract]). 

The following keywords were used in the PsycINFO and Embase searches: Panic AND (watch OR wearable OR device* OR ambulatory) AND ((‘heart rate’ OR hrv OR respir* OR breath* OR cardiac OR cardioresp* OR cardiac OR ‘tidal volume’ OR variability)). 

The reference lists of relevant studies and pertinent review articles were also used to retrieve additional research.

#### 2.1.2. Inclusion and Exclusion Criteria 

Studies were included in the review if they met the following criteria: participants ≥ 18 years old; inclusion of a primary diagnosis of PD with or without AG; provision of respiratory and/or cardiac measurements obtained outside of the laboratory through wearable technology devices (hereafter referred to collectively as “wearables,” i.e., smart non-invasive electronic devices that can be physically worn by individuals without encumbering daily activities or restricting mobility, such as accessories, biopatches, or objects embedded in clothing, which automatically collect, monitor, analyze, and communicate personal data [27]); written in English; and availability of full text. Conference papers and letters were included only if they reported exhaustive data. Studies were excluded if they did not provide separate results in subgroups with PD when multiple anxiety disorders were studied or if they provided respiratory and/or cardiac measurements obtained through wearables in the laboratory only. Reviews, meta-analyses, case reports, book chapters, editorials, and conference abstracts were also excluded. 

#### 2.1.3. Screening and Extraction of Data 

Three of the study’s authors (S.D., D.D.D, and D.C.) independently carried out search and screening process; inconsistencies were discussed and resolved before proceeding. 

**Figure 1:**
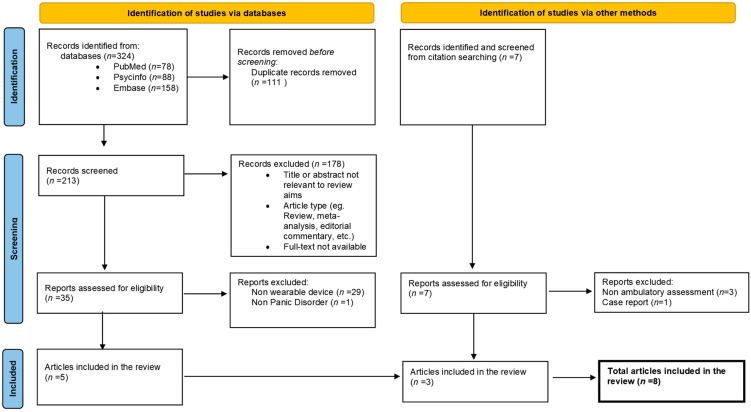
PRISMA flow diagram.

Two hundred and twenty records were screened to yield eight suitable articles in this review (Figure 1, PRISMA flow diagram). 

#### 2.1.4. Risk-of-Bias Assessment 

Finally, in the framework of the PRISMA statement [26], an assessment of the risk of bias across all of the reviewed studies was conducted. In addition to the critical aspects specifically related to the use of wearables, we considered other methodological domains suitable for evaluation in the reviewed studies, considering that they presented strong heterogeneity in terms of design and aims and, in some cases, were secondary analyses of sub-samples from larger studies with different aims. We mainly focused on the following: sampling bias, including biases resulting from recruitment strategies and inclusion/exclusion criteria used; reporting bias, due to selective information or outcome reporting and/or non-reporting of relevant information or outcomes that would have been expected to be reported; and other biases, including biases resulting from power calculation. Review authors’ judgments were categorized as “low risk” of bias, “high risk” of bias, or “some concerns” of bias (i.e., when a possible risk was present, but at a lower level than “high risk”). The assessment of the risk of bias was performed independently by two authors (D.C. and S.D.), and inconsistencies in the results were discussed and resolved. Based on the methodological evaluation, a judgment about the quality of the reviewed studies was also provided. 

### 2.2. Results 

The eight articles fitting the inclusion criteria are detailed in Table 1. Three of them presented different analyses from a study performed with the LifeShirt system [16,17,28], two articles were conference papers that presented different aspects of the same study carried out with the Zephyr BioPatch [29,30], and the other three articles presented studies performed with different smartwatches, namely the Polar RS800CX [31], the Garmin Forerunner 310 XT [32], and the Garmin Vivosmart 4 [33]. The studies using the LifeShirt system [16,17,28] and the Zephyr BioPatch [29,30] obtained both respiratory and cardiac measurements, while the three studies using the smartwatches [31,32,33] provided cardiac measurements only. Finally, five of the studies recorded physiological measurements during the participants’ daily lives [16,17,28,29,30,33], while the other two [31,32] contained measurements captured during in vivo exposure therapy. 

In the following paragraphs, we succinctly describe the main features of the different types of wearables and the main results concerning cardiorespiratory measurements obtained from the various studies. 

**Table 1:** Details of the selected studies.

Authors, Year [Ref.]	Pfaltz et al., 2009 [17]	Pfaltz et al., 2010 [16]	Pfaltz et al., 2015 [28]	Rubin et al., 2015 [30]; Cruz et al., 2015 [29]	White et al., 2017 [32]	Mumm et al., 2019 [31]	Tsai et al., 2022 [33]
**Study design**	Cross-sectional comparison study between patients with PD and HCs. Single site	As in Pflatz et al., 2009 [17]	As in Pflatz et al., 2009 [17]	Longitudinal open study (detection-feasibility study). Single site	Sub-analyses of a selected sample from a multicenter longitudinal randomized treatment study #	Secondary analysis of a multicenter randomized controlled study investigating the augmenting effect of DCS compared to a placebo on 12-session CBT with in vivo exposure [34]	Longitudinal open study (development study of a PA ML prediction model). Single site
**Participants, recruitment, and psychiatric assessment methodology**	N = 26 patients with PD (85% F; mean age: 35.5 ± 10.9 years) and 26 HCs (77% F; mean age: 37.1 ± 9.6 years), recruited via local newspaper advertisements. Structured Diagnostic Interview for Mental Disorders (DSM-IV)	As in Pflatz et al., 2009 [17]	N = 19 patients with PD (84% F; mean age: 32.8 ± 9.6 years) and 20 HCs (80% F; mean age: 35 ± 8.3 years) who were selected from the original sample in Pfaltz et al., 2009 [17], based on the completeness of data recorded	N = 10 participants (5 females, 4 males, 1 transmale) recruited from local Meetup groups, Google AdWords, and the website, Craigslist. Each participant self-identified him/herself as suffering from PD	N = 85 patients with PD and AG (59% F; mean age: 33.89 ± 10.51; 43 patients were in the standard in vivo exposure condition, while the others were in the augmented exposure condition). The patients were selected based on data availability from a larger sample # recruited through physician referral and advertisements in media outlets. The DSM-IV TR Composite International Clinical Diagnostic Interview	A subgroup of N = 27 outpatients (from a specialized clinic for anxiety disorders) with AG (with or without PD) § with available HR and HRV recordings during in vivo exposure. No information on sex distribution or age of this subgroup. Clinical diagnosis based on ICD-10 criteria	N = 59 patients with PD (61% F; mean age: 46.2 ± 14.7), recruited from patients referred to a single Hospital in Taiwan. The DSM 5 clinician-administered Mini International Neuropsychiatric Interview
**Comorbid psychiatric diagnoses. Current medications (number of participants)**	AG = 23; MDD = 4; PTSD = 3; social phobia = 3; hypochondria = 1; SSRIs = 7; Benzodiazepines = 4; Analgesic drugs = 3. Exclusion criteria were medical diseases or medications possibly influencing cardiorespiratory functions	As in Pflatz et al., 2009 [17]	AG = 19; MDD = 2; Social phobia = 2; primary insomnia = 1. SSRIs = 4; Benzodiazepines = 2; Noradrenergic and serotonergic antidepressant = 1; Angiotensin II receptor antagonist = 1	No information available	Exclusion criteria were comorbid psychotic or bipolar I disorder, substance-use disorder, current psychotherapeutic or psychotropic interventions (as screened in the original study #). Medical illnesses (e.g., cardiovascular or neurological) that excluded exposure-based CBT. No information about medications possibly influencing cardiac function	In total, 31 (42%) patients from the entire original sample were taking psychopharmacotherapy (stable for at least 4 weeks; no changes allowed during the study). Patients with severe medical diseases, cardiovascular diseases, or taking medications possibly influencing cardiac function were excluded from the secondary analysis	In total, 51% of patients had at least 1 comorbid psychiatric disorder (mainly AG, GAD, MDD, and PTSD). Current substance-use disorder and “cardiopulmonary incapacity” were among exclusion criteria. No information about any types of medication
**Setting**	Daily life	Daily life	Daily life	Daily life	In vivo exposure therapy (bus rides, suitable for HR collection due to the relatively minor bodily movements). Patients had to assume a seated position to minimize artifacts	Personalized in vivo exposure therapy (e.g., public transportation, elevators, driving cars, etc.)	Daily life
**Duration of the study**	Two 24-h recordings one week apart. Analyses were restricted to data recorded during waking periods (9 a.m.–9 p.m.)	As in Pfaltz et al., 2009 [17]	As in Pfaltz et al., 2009 [17]	Three weeks	The entire treatment comprised 12 CBT sessions and 2 follow-up booster sessions (2 and 4 months later). The sample included in this study completed a total of 233 bus-based exposure exercises	Duration of the entire study: 4 months. Exposure-related HR and HRV (whose aim is relevant to the present review) § were evaluated only during the first exposure in the first exposure session because it was the only exposure without DCS or placebo.	One year
**Aim**	To assess respiratory patterns in patients with PD during physical inactivity *, in comparison with HCs	To assess respiratory pattern in patients with PD compared with HCs using respiratory data stratified for predefined levels of physical activity *	To investigate whether patients with PD presented HR acceleration higher than the metabolic demand (metabolic decoupling, MD) and whether MD was related to phasic and tonic anxiety. The MV and AccM-physical activity were used to index metabolic demand	Proof-of-concept of panic-attack prediction based on physiological data (HR, BR, HRV, Temp). The predictive models were developed by using change-point analysis *** and anomaly-detection algorithms	To cluster the HR responses during exposures (by latent class cluster analysis of the individual raw HR data); to examine changes in intra-individual HR-cluster membership across sessions, and associations between HR-response types and panic-related symptoms	Aim, relevant to the present review: To evaluate HR and HRV changes during the first exposure in the first exposure session §	To build a 7-day PA ML learning prediction model using multiple physiological, clinical, and environmental potential predictors continuously collected during daily life
**Type of wearable**	LifeShirt system (Vivometrics Inc., Ventura, CA, USA)	LifeShirt system (Vivometrics Inc., Ventura, CA, USA)	LifeShirt system (Vivometrics Inc., Ventura, CA, USA)	Zephyr BioPatch (Medtronic, Inc., MN, USA)	Garmin Forerunner 310 XT (Garmin Ltd., Southampton, UK)	Polar RS800CX and accelerometer (Polar Electro Oy, Kempele, Finland)	Garmin Vivosmart 4 (Garmin International, Inc., Olathe, KS, USA)
**Cardiorespiratory measures**	TV, Ttot, MV, f/TV, TV/Ti, Ti/Ttot, Sighs, Sighs%; SD and RMSSD of TV, MV, and Ttot were calculated as indices of respiratory variability	TV, MV, Ttot, Sighs, Sighs%. RMSSD of TV and MV were calculated as indices of respiratory variability	HR, MV. Within-individual pairwise Pearson correlations of minute-by-minute average r (H-Acc), r(H-MV), and r(HR-Acc, MV) were calculated as univariate indices of metabolic coupling	HR, HRV, BR.	HR. Baseline was defined as the tonic, pre-boarding HR levels. Data were segmented into 3 epochs: before, during, and after exposure exercise	HR and two HRV indices (i.e., RMSSD of the NN intervals and HF), which reflect parasympathetic nervous system activity. Only movement-free five-min intervals were used. The HRV indices were calculated using the software, Kubios HRV	Minimum and maximum HR, average HR (during the past 7 days), and the average HR at rest, all in bpm
**Other physiological or environmental measures of interest**	AccM	AccM	AccM	Temp, AccM	GPS location, speed	AccM	Wakefulness and total-, deep-, light-, and REM-sleep duration; floors climbed, distance traveled, steps taken; multiple indices of air quality
**Clinical measures**	Psychometric questionnaires before recordings: RSQ, PDSS, MI, STAI, ASI, BDI. During recordings: self-reported PA occurrence (through a customized electronic diary)	Psychometric questionnaires before recordings: RSQ, PDSS, MI, STAI, ASI, BDI. During recordings: self-reported anxiety every three hours (by a customized electronic diary)	Tonic anxiety: STAI-Trait and ASI before recording; the mean diary-reported anxiety levels during recordings. Phasic anxiety: standard deviation of diary-reported anxiety levels	Self-reported PAs, by a smartphone application	Pre-therapy assessment: MI, BSQ, ACQ; during exposure: self-reported anxiety collected by EMA device	_	Self-report clinical measures (BDI-II, BAI-II, STAI-S, STAI-T, PDSS-SR) were collected at 2-week intervals, through a mobile app. “PA yes” was defined as scores from 1 to 5 for the first question of the PDSS-SR; “PA no” was defined as scores of 0
**Main results**	The two groups did not differ in terms of age, gender, body-mass index. No significant respiratory differences between PD group (with or without current medications) and HCs (alpha level was set at 0.05)	No significant respiratory differences between PD group and HCs (alpha level was set at 0.05), except for higher variability (RMSSD) of TV in PD group vs. HCs (*p* = 0.04) during minimal movement and slow walking.	The rHR-Acc, rHR-MV, and rHR-(Acc, MV) were lower (*p* = 0.005, 0.009, and 0.002, respectively) in PD group than in HCs, indicating metabolic decoupling in PD, even considering physical-fitness indices ** as covariates. In PD group, HR–Acc coupling was inversely related to ASI (*p* = 0.02) and phasic daytime anxiety (*p* = 0.047) (periods with PAs were excluded; alpha level was set at 0.05)	Seven out of ten participants were analyzed. Accounting for AccM-based physical activity, predictive models identified that in pre-panic periods, HR, BR, and Temp were higher and HRV was lower than expected, compared with non-panic periods. Only general descriptive statistics were reported. No statistics concerning the significance and accuracy of the predictive model were provided.	Pre-treatment-scale scores indicated that patients had moderately severe symptoms. Three low-level clusters (3, 4, 6) with a median HR < 91 bpm and four high-level clusters (1, 2, 5, 7) with a median HR > 97 bpm were identified. Clusters 5 and 7 presented increased HR variability and greater pre-boarding HR changes than the others. Low and relatively unvarying HR responses (e.g., cluster 4) were associated with better tolerance of bodily symptoms (*p* = 0.02) and low self-reported anxiety (*p* = 0.001) during exposure.	Significant increase in both HRV indices (HF and RMSSD of NN, *p* = 0.02 and 0.007, respectively) from the beginning to the end of the exposure, indicating higher parasympathetic activity at the end of the exposure (alpha level was set at <0.05)	The Random Forest ML method provided the best performance (accuracy = 81.3%) in predicting PAs one week before their occurrence. Main predictors: BAI-II, BDI-II, and STAI-S and -T scores; Mini International Neuropsychiatric Interview; average and resting HR; and deep-sleep duration (no specifications about values or directionality of predictors were reported)
**Other findings of interest**	In PD group, RSQ scores were correlated negatively with f/TV (*p* = 0.006) and positively with Ttot and RMSSD of Ttot. (*p* = 0.01 and 0.05, respectively)	Self-reported anxiety levels during daily life recordings were higher in PD group than HCs (*p* < 0.001). The two groups did not differ in time spent at different physical activity levels.	Anxiety levels were higher in PD group than HCs (*p* < 0.001). The two groups did not differ in mean accelerometer measures, MV, and physical fitness indices **.	The PA-related symptoms with highest average severity among participants were anxiety, worry, and shortness of breath; those with lowest severity were hot/cold flashes and fear of dying.	Self-reported anxiety declined across sessions (*p* = 0.002). The HR-cluster assignments of each participant were not stable across sessions or treatment conditions. Female patients were more commonly assigned to high-variability HR clusters (2, 5, and 7)	During exposure, patients with psychopharmacotherapy presented significantly (*p* < 0.01) reduced HF HRV and RMSSD of NN than those without psychopharmacotherapy	The prediction performance of the all-feature model was better than that of the physiological–environmental model (accuracy = 0.67%) and the questionnaire model alone (accuracy = 0.77%)

ASI = Anxiety Sensitivity Index; AccM (units) = accelerometry (motion); ACQ = Agoraphobic Cognition Questionnaire; AG = agoraphobia; bpm = beats per minute; BAI = Beck Anxiety Inventory; BDI = Beck Depression Inventory; BSQ = Body Sensation Questionnaire; CBT = cognitive-behavioral therapy; DCS = D-cycloserine; DSM-IV = Diagnostic and Statistical Manual of Mental Disorders, 4th Edition (1994); EMA = ecological momentary assessment with a handheld computer and customized software; F = females; f = frequency of breathing (bpm, breaths per minute); f/TV (bpm/L) = an index of rapid shallow breathing; GAD = generalized anxiety disorder; GPS = Global Positioning System; HCs = healthy controls (i.e., without any lifetime anxiety disorders or current psychiatric disorders); HF = high-frequency band; HR = heart rate; HRV = heart-rate variability; ICD-10 = International Statistical Classification of Diseases and Related Health Problems, 10th revision; MDD = major depressive disorder; MI = Mobility inventory for agoraphobia; ML = machine learning; MV (L/min) = minute ventilation; N = number of participants; NN = RR interval (i.e., the time between each detected heartbeat) after removing artifacts and noise; PA(s) = panic attack(s); PD = Panic Disorder; PDSS = Panic Disorder Severity Scale; PDSS-SR = Panic Disorder Severity Scale—Self-Reported; PTSD = post-traumatic stress disorder; REM = rapid-eye movement; RMSSD = root mean square of successive differences; RSQ = Respiratory Symptoms Questionnaire; SD = standard deviation; SSRIs = selective serotonin reuptake inhibitors; STAI = State (S)-Trait (T) Anxiety Inventory; Temp = core body temperature; Ti (sec) = inspiration-breath time; Ttot (sec) = 60/frequency of breathing per min; Ti/Ttot (ratio) = duty cycle, indexing the timing of the respiratory on/off switch; Sigh = inspiratory-breath volumes > 2.5 times the median of running baseline of TV over 2-min duration; Sighs% = the total number of sighs divided by the total number of breaths; TV (mL) = tidal volume; TV/Ti = mean inspiratory flow, a putative measure of respiratory drive; * Six physical-activity categories (i.e., inactivity, minimal movement, slow walking, moderate walking, fast walking, and running) assumed to be relevant in daily life were established based on the quiet-sitting and paced-walking measurements conducted in the laboratory, before starting the recordings in the natural environment. Through customized programs, mean accelerometer motion (AccM) boundaries for each activity category were calculated. Each breath was classified as belonging to the inactivity category, or one of the others, by searching through the AccM signal, using the previously determined category boundaries.** A composite individual physical-fitness index was calculated, including participants’ sex and slope and intercept from the linear prediction of HR by means of accelerometery during a task performed before leaving the laboratory (i.e., sitting quietly for 2 min and then walking at slow, intermediate, and fast standardized paces for 3 min). *** The change-point analysis identifies locations where significant changes occur in time-series data. # This is a subsample of a randomized multicenter-treatment study comparing standard in vivo exposures to fear-augmented exposures (i.e., standard exposure with additional interoceptive exposure); in turn, the multicenter-treatment study is a second phase of a 6-month multicenter randomized controlled trial [35]. § This is a subgroup of a larger sample included in the study by Mumm et al. [31], whose general aim of evaluating changes in HR and/or HRV after CBT were beyond the aims of this review, as their findings were recorded in a laboratory.

#### 2.2.1. LifeShirt-System-Based Results 

The LifeShirt system (Vivometrics Inc., Ventura, CA, USA) is a FDA-approved multi-channel ambulatory monitoring instrument capable of continuously recording several physiological signals, including multiple respiratory measures, electrical heart activity (electrocardiogram, ECG), O_2_ saturation, and posture and activity levels, for up to 24 h. The system consists of a Lycra undergarment vest with embedded respiratory-inductive plethysmography (RIP) and various additional sensors, a portable digital recorder, and a PC-based analysis software (Vivologic). The RIP monitors respiration through two inductive coils placed around the rib cage (RC) and abdomen (AB). This functions as an indirect method to assess respiratory variables and estimate lung volume from respiratory movements based on two degrees of freedom (DOF; i.e., with two independent variables), wherein the tidal volume (TV) is assumed to be equal to the sum of the RC- and AB-volume changes. The RIP captures circumferential changes in the RC and AB during breathing as the raw voltage changes. To obtain respiratory-volume data, the raw data captured by the LifeShirt system must be converted to liters through user-specific calibration procedures [21,36,37]. In studies on the LifeShirt system [16,17,28], calibration was a two-step process. First, the proportional relationship of the RC vs. AB displacements to the TV were estimated for each participant by using a standard qualitative diagnostic calibration during a 5-min quiet-sitting period. Second, the two respiratory waveforms from the RC and AB bands (sampled at 50 Hz) were summed and converted to absolute TV in mL by a fixed-volume procedure of breathing in and out of a 750-milliliter plastic-bag system eight times in a sitting position. 

The cardiac activity was assessed through three electrodes placed on the upper chest and lateral abdominal surface, sampled with a lead-II ECG (200 Hz). Finally, the LifeShirt system monitors physical activity through a tri-axial accelerometer embedded at the sternum level. 

Of the three articles presenting LifeShirt-system-based results (Table 1), two [16,17] were intended to evaluate whether the patients with PD presented respiratory-pattern abnormalities during their everyday lives that were similar to those that were repeatedly found in the laboratory. They provided different analyses of the same sample of 26 patients with PD and 26 HCs free from cardiovascular and respiratory disease and medications with relevant autonomic or respiratory effects. The expected respiratory abnormalities in the patients with PD were not found during the periods of physical inactivity during daytime wakefulness [17], while the analyses of different physical activity levels revealed significantly increased tidal-volume variability in the patients with HCs only during minimal movement and slow walking [16]. Therefore, little support was provided for previous laboratory-based respiratory findings for PD. 

The third article [28] (Table 1) focused on the comparison of metabolic-regulation mechanisms in a subgroup of 19 patients with PD and AG and 20 HCs selected from the original sample mentioned above. Given that a large part of HR variation during daily life usually reflects metabolic demand (metabolic coupling), the authors hypothesized that patients with PD may have an additional anxiety-related contribution to HR, resulting in HR acceleration that is greater than metabolic demand (metabolic decoupling). Accelerometer measurements and minute ventilation were used as metabolic-activity markers to index metabolic contributions to HR. Self-reported levels of phasic and tonic anxiety were noted in an electronic diary. As expected, the PD group exhibited significantly decreased coupling of their metabolic-activity markers from their HRs compared to their HCs, and significant associations between metabolic decoupling and some measures of anxiety were found.

#### 2.2.2. Zephyr-BioPatch-Based Results 

The Zephyr BioPatch (Medtronic Inc., Minneapolis, MN, USA) is a chest-worn patch-style device consisting of a central electronic module (BioModule) that snaps into the BioModule Holder, which is attached to the subject via disposable standard ECG electrodes. The BioModule must be placed in the epigastric quadrant of the subxiphoid region and can transmit physiological data to and/or record the data in its internal memory. Through proprietary circuitries and algorithms, the device continuously records multiple physiological and biomechanical signals to generate relevant outputs, such as beat-to-beat “R to R”, HR per minute, heart-rate variability (HRV), BR per minute, tri-axis accelerometer-based posture and activity, estimated core body temperature, and calories. The BR is estimated by impedance pneumography (IP) on the BioPatch, with the signal passing through the electrodes that are used for the ECG. According to the IP, the individual’s BR is determined by the changing impedance, measured as voltage changes in the electrodes, of the thoracic cavity, which increases during inhalation and decreases during exhalation [38]. In addition to algorithm-based outputs, raw data from the recordings can also be downloaded. The Zephyr BioPatch has a sampling frequency of 250 Hz for ECG, 25 Hz for BR, and 100 Hz for tri-axis acceleration. It reports HR and BR every second with millisecond precision. 

The study using the Zephyr BioPatch aimed to develop a system combining a personal wearable device with a smartphone application, with the ultimate goal of predicting the occurrence of PAs based on physiological data and delivering in-the-moment mobile-based interventions, such as breathing and relaxation exercises. Two conference papers described herein [29,30] (Table 1) presented the first steps in this project, namely the preliminary results from an initial 3-week detection-feasibility study conducted on a small real-world data set from 10 participants. The HR, BR, HRV, and core body temperature were used as the inputs within supervised anomaly-detection algorithms to predict imminent PAs, thereby providing a binary output classified as either “pre-panic” or “non-panic.” In line with the aim of the study, each of the above measurements was summarized at a frequency of 1 Hz. The reporting of the occurrence of PAs in daily life, which was required to train and validate the prediction model, was undertaken manually by each participant through a smartphone application. The preliminary results of the PA detection revealed variations in physiological parameters that could identify “pre-panic” periods. No statistics were provided concerning the accuracy of the panic prediction. 

Overall, the prototype of the mobile application was intended to report current physiological parameters to users, notify the occurrence of physiological modifications that might be related to an imminent PA (“pre-panic” periods), and provide graphical interfaces for breathing- or relaxation-exercise interventions. 

#### 2.2.3. Smartwatch-Based Results 

The three remaining articles [31,32,33] (Table 1) presented results obtained through three different types of smartwatch to record HR [31,32,33] and HRV [31] in various contexts.

White et al. [32] (Table 1) clustered the HR responses of patients with PD and agoraphobia who undertook repeated situational exposure (i.e., rides on a bus) to determine how HR-response types related to PD symptoms. This study was a secondary analysis of 85 patients selected, based on data availability, from the second phase of a 6-month multicenter randomized controlled trial [35]. The original aim of the trial was to evaluate whether therapist-guided exposure (T+) in situ (12-session manualized cognitive-behavioral therapy (CBT)) was associated with greater clinical effectiveness than therapist-prescribed exposure (T-) in situ (12-session manualized CBT). Three hundred and sixty-nine patients were randomized to CBT T+, CBT T+, or wait-list control group [35]. As declared by White et al. [32], the second phase compared two randomized active treatments, namely standard situational exposure and fear-augmented exposure (i.e., standard exposure combined with interoceptive exposure). However, details on this phase were lacking, even in the reference provided by the authors [39]. 

The authors used the wrist-worn smartwatch, Garmin Forerunner 310 XT (Garmin Forerunner 310XT, Garmin Ltd., Southampton, UK), a commercial monitor capable of collecting locations, through global positioning system (GPS) coordinates, and HR, through electrodes placed on the back of a chest strap, worn just below the breastplate. The authors identified seven interpretable clusters. Overall, the clusters with low and relatively stable HRs before and during exposure were associated with lower self-reported anxiety and better bodily symptom tolerance, while the opposite was found for the clusters characterized by higher absolute levels and larger fluctuations in HR. The individual HR responses were dispersed across the clusters, suggesting that extrinsic environmental factors provided an additional contribution, beyond intrinsic personal factors, to the shaping of the response characteristics.

A similar device was used by Mumm et al. [31] (Table 1) to record the HRs and HRVs in a sample of 66 patients with AG with and without PD who undertook manualized CBT. The authors evaluated possible changes in HR and/or HRV after the CBT. The study utilized the Polar RS800CX (Polar Electro Oy, Kempele, Finland), a wrist-worn smartwatch capable of recording cardiac activity by using electrodes placed on the back of a chest strap, and connected to an accelerometer. This study was a secondary analysis of a 4-month randomized controlled trial investigating the augmenting effect of D-cycloserine (DCS) compared to a placebo on 12-session CBT with in vivo exposure [34]. For the purpose of this review, we only examined results concerning the HRs and HRVs recorded during in vivo exposure in a subsample of 27 patients and the data from the first exposure in the first exposure session only, as this was the only exposure without the influence of DCS or placebo (all the other pre- and post-CBT cardiac recordings were conducted in the laboratory). Only the movement-free periods during the exposure were used, to minimize the confounding influences of motion on the cardiac measurements. Overall, the results suggested an increase in parasympathetic nervous system activity at the end of the exposure.

Finally, Tsai et al. [33] (Table 1) used the wrist-worn smartwatch, Garmin Vivosmart 4 (Garmin International, Inc., Olathe, KS, USA), to continuously collect physiological data, including HR, activity levels, and the duration of different sleep stages, during a 1-year real-life study on 59 patients with a primary diagnosis of PD. The Garmin Vivosmart 4 provides continuous monitoring of HR through photoplethysmography (PPG), a non-invasive optical measurement method that uses a light source and a photodetector on the surface of the skin to measure volumetric variations in blood circulation [40,41]. The device also captures the steps taken, distance traveled, floors climbed, duration of wakefulness, and different sleep stages of its wearers. The study aimed to provide a 7-day PA machine learning (ML) prediction model using a series of potential predictors collected over the course of one year. The potential predictors included the above-mentioned physiological data, the clinical features obtained through repeated self-report psychometric questionnaires (administered through an internet-based mobile application), and multiple indices of air quality obtained from a local open-environmental-data platform. The prediction model’s ground truth (i.e., the label “PA yes” versus “PA no” in the previous week) was based on the first question on a 5-point scale (scored from 0 = not at all, to 5 = extreme), the Panic Disorder Severity Scale-Self Report (i.e., “How many panic and limited-symptom attacks did you have during the week?”). The response “PA yes” was assigned to scores from 1 to 5, whereas “PA no” was assigned to the “0” scores. The same sample of patients was used for both training and testing the ML models by using data sets from approximately the first 10 months and the last two months of the study, respectively. Of the six different ML models tested, the Random Forest model offered the highest accuracy in the prediction of PAs one week before their occurrence. Some of the clinical measures, average and resting HR and deep-sleep duration, were the most important variables in the predictive model. 

#### 2.2.4. Risk of Bias and Quality of the Reviewed Studies 

**Figure 2:**
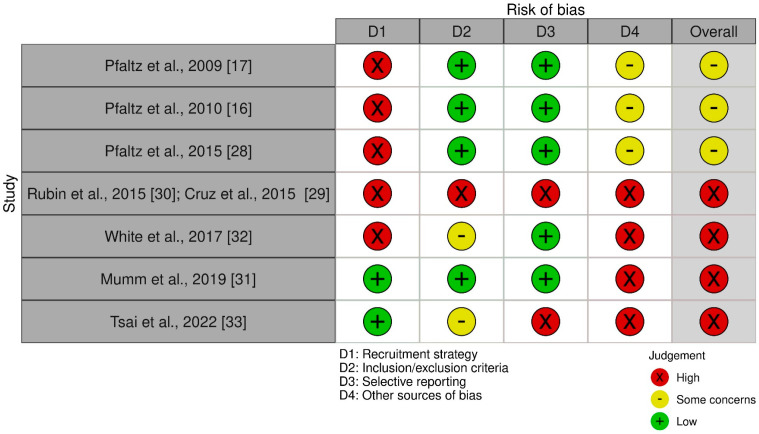
Graphical presentation of risk of bias in the individual selected studies [16,17,28,29,30,31,32,33].

A graphical presentation and details on the risk of bias in the individual selected studies are presented in Figure 2 and Table 2, respectively. In terms of selection bias, biases in the recruitment strategies and the inclusion/exclusion criteria used were present, possibly undermining the validity and/or generalizability of the results. In the study performed with the LifeShirt system [16,17,28], although the psychiatric and medical assessment was rigorous and presented a low risk of bias in the inclusion/exclusion criteria, the recruitment strategy increased the risk that the results would not be generalizable to clinical populations with PD. Furthermore, as the three reviewed LifeShirt-system-related articles [16,17,28] were based on re-analyses of the same sample, the certainty of the evidence was low. The sample in the study performed with the Zephyr BioPatch [29,30] presented a high risk of bias concerning both the recruitment and the inclusion/exclusion criteria. A high risk of recruitment-related bias was also present in the study with the Garmin Forerunner 310 XT [32]. Two of the three smartwatch-based studies raised some concerns related to possible bias in the inclusion/exclusion criteria [32,33]. 

In two studies [29,30,33], a high risk of reporting bias was present. 

A power calculation was performed in the LifeShirt-system study [16,17,28] in order to detect large effect sizes, but this raised the concern that small-to-moderate differences were not detected between patients with PDs and HCs. The use of a power calculation was lacking in the study by Mumm et al. [31], and it was not appropriate in the other studies.

In both PA-prediction studies [29,30,33], the use of the self-reported experience of PAs conferred a high risk of inappropriate labeling upon the predictive model’s development. This risk was especially high in the Zephyr-BioPatch–based predictive study [29,30], as the authors included symptoms, such as anxiety and worry, that are not included in the current nosographic criteria for defining PAs. 

The lack of HCs in the studies assessing HR and HRV during exposure situations [31,32] raised concerns about the specificity of results. Finally, all the critical methodological aspects specifically related to the use of wearables are extensively described and considered in the Discussion section. 

Overall, due to the several limitations of the body of the reviewed studied, the certainty of the evidence is generally low and improvements in quality are required in future studies.

**Table 2:** Details of the risk of bias in the individual selected studies.

Authors, Year [Ref.]	Selection Bias		Reporting Bias	Other Sources of Bias		
	*Recruitment Strategy*	*Inclusion/Exclusion Criteria*	*Selective Reporting*	*Power Calculation*	*Labeling Methodology in PA Prediction Studies*	*Adjunctive Bias*
**Pfaltz et al., 2009 [17]**	Local newspaper advertisements (H)	Appropriate criteria assessed by clinician-administered psychiatric interview and medical examination. Exclusion criteria related to medications possibly influencing cardiorespiratory functions were present (L)	Relevant information appropriately reported (L)	Power calculation performed. Study powered to detect large effect sizes; possibly missed small–moderate effects (SCs)	_	_
**Pfaltz et al., 2010 [16]**	Local newspaper advertisements (H)	As in Pfaltz et al., 2009 [17] (L)	Relevant information appropriately reported (L)	As in Pfaltz et al., 2009 [17] (SCs)	_	_
**Pfaltz et al., 2015 [28]**	Local newspaper advertisements (H)	As in Pfaltz et al., 2009 [17] (L)	Relevant information appropriately reported (L)	As in Pfaltz et al., 2009 [17] (SCs)	_	_
**Rubin et al., 2015 [30]** **; Cruz et al., 2015 [29]**	Local Meetup groups, Google AdWords, and the website, Craigslist (H)	Self-reported PD (the sole inclusion criterion); no exclusion criteria (H)	Incomplete reporting of sample features and results. No statistics concerning the results and predictive model were provided (H)	Power calculation was not appropriate for the study	Self-reported experience(s) of PA(s) (H)	_
**White et al., 2017 [32]**	Mixed recruitment strategy, including physician referral and advertisements in media outlets (H)	Appropriate diagnosis-related criteria assessed by clinician-administered psychiatric interview. Medical-disease-based exclusion criteria were present. Lack of exclusion criteria related to medications possibly influencing cardiac function (SCs)	Relevant information appropriately reported (L)	Power calculation was not appropriate for the study	_	Lack of healthy control group (H)
**Mumm et al., 2019 [31]**	Selection of patients referred to a specialized clinic for anxiety and related diseases (L)	Appropriate diagnosis-related criteria assessed by clinician-administered psychiatric interview. Exclusion criteria related to medical diseases and medications possibly influencing cardiac function were present (L)	Relevant information appropriately reported (L)	Power calculation was lacking (H)	_	Lack of healthy control group (H)
**Tsai et al., 2022 [33]**	Selection of patients referred to a hospital (L)	Appropriate diagnosis-related criteria assessed by clinician-administered psychiatric interview. Lack of medication-related exclusion criteria and definition of the exclusion criterion, “cardiopulmonary incapacity” (SCs)	No reporting of current medications, values or directionality of predictors (H)	Power calculation was not appropriate for the study	Self-reported experience(s) of PA(s) (H)	_

L = low risk of bias; H = high risk of bias; PA(s) = panic attack(s); PD = panic disorder; SCs = some concerns over risk of bias.

## 3. Part 2: Pilot Comparison of Simultaneous Cardiorespiratory Recordings by the Wearable Zephyr BioPatch and the Quark-b2 Stationary Testing System 

### 3.1. Materials and Methods

In this pilot study, we evaluated the accuracy of the chest-worn wearable Zephyr BioPatch (Zephyr BioPatch, Medtronic, Inc., Minneapolis, MN, USA) in estimations of HR and BR compared with the Quark-b2 stationary testing system (Cosmed, Italy). The Zephyr BioPatch is described above, in Section 2.2.2. For additional details on the Materials and Methods, please see the Supplementary Materials. 

Briefly, the Quark-b2 stationary testing system assesses respiration physiology on a breath-by-breath basis. It is a validated instrument widely used in sports medicine and respiration-physiology studies [42]. An open, light face mask connects the subject to the respiratory testing system. The Quark-b2 also assesses cardiac activity using a 12-lead ECG monitor to provide HR and HRV estimation. The system reports BR, multiple respiratory volumetric measures, and HR, for every breath, to the nearest second.

We simultaneously recorded respiratory and cardiac signals by applying the Zephyr BioPatch, placed in the epigastric quadrant of the subxiphoid region, and the Quark-b2 stationary testing system, to 10 volunteers (5 males and 5 females) recruited from among staff of Villa San Benedetto Menni Hospital, Albese con Cassano, Como, Italy. Recordings were taken over 20 min and captured when volunteers were at rest and in a sitting position. Beyond obtaining written informed consent from the participants, no inclusion or exclusion criteria were applied. 

The experimental setup and the two instruments are shown in Figures S1 and S2 in the Supplementary Materials. 

We compared the performances of the two devices using Bland–Altman (B&A)-plot analysis [43,44,45,46], which quantifies the agreement between two quantitative methods of measurement by studying the mean difference and constructing limits of agreement. We also applied linear mixed model (regression) analyses to investigate whether the estimated biases of the two devices were different at different levels of measurement, and whether they were affected by age, sex or body-mass index. We set the level of significance at the conventional value of 0.05 and performed the analyses using the R programming language, version 3.6.3 *(R Core Team, R: A Language and Environment for Statistical Computing, R Foundation for Statistical Computing, Vienna, Austria, 2020*). 

### 3.2. Results

The full results of our pilot study are detailed in the Supplementary Materials.

We obtained 176 and 175 matched 1-min-data-collection periods for the HR and BR, respectively, from the simultaneous recordings provided by the two devices. 

In the B&A-plot analysis of the 1-min-average HR (Figure S3 in the Supplementary Materials), no significant overall bias across the two devices was found. However, a statistically significant variation in the bias at different average HR values (B = 0.539, *p* < 0.001) was detected. The predicted bias at the minimum observed 1-min-average HR value (60.342) was −1.046, indicating the expected overestimation of the Zephyr BioPatch compared to the Quark-b2. The predicted bias at the maximum observed 1-min-average HR value (101.81) was 1.912, indicating the expected underestimation of the Zephyr BioPatch compared to Quark-b2. The upper- and lower-bound limits of agreement were 2.044 (95% bootstrap confidence intervals: 1.506; 2.413) and −1.812 (95% bootstrap confidence intervals: −2.109; −1.373), respectively. 

Similarly, no significant overall bias was found by analyzing the 1-min-average BR, whereas a statistically significant variation in the bias at different average BR values (B = 0.10283, *p* < 0.001) was found (Figure S5 in the Supplementary Materials). The predicted bias at the minimum observed 1-min-average BR value (5.431) was −5.1, indicating the expected overestimation of the Zephyr BioPatch compared to the Quark-b2. The predicted bias at the maximum observed 1-min-average BR value (24.45) was 4.123, indicating the expected underestimation of the Zephyr BioPatch compared to the Quark-b2. The upper- and lower-bound limits of agreement were 3.705 (95% bootstrap confidence intervals: 2.617; 4.584) and −3.51 (95% bootstrap confidence intervals: −4.145; −2.661), respectively (Figure S5 of the Supplementary Materials).

Finally, when we used the breaths detected by the Quark-b2 instead of the 1-min episodes as a time unit, the B&A-plot analysis revealed a significant overall bias in both the HR (Figure S4 in the Supplementary Materials) and the BR (Figure S6 in the Supplementary Materials), indicating an average overestimation by the Zephyr BioPatch compared to the Quark-b2. A statistically significant variation in the bias at different average values of both parameters was also found. The predicted biases at the minimum observed breath-by-breath Quark-b2 HR and BR measurements were −5.155 and −11.844, respectively, indicating the expected overestimation of the Zephyr compared to Quark-b2. The predicted biases in the maximum observed breath-by-breath Quark-b2 HR and BR measurements were 16.65 and 31.024, respectively, indicating the expected underestimation of the Zephyr compared to the Quark-b2. The upper- and lower-bound limits of agreement for the HR were 6.352 (95% bootstrap confidence intervals: 5.886; 6.897) and −5.9287675 (95% bootstrap confidence intervals: −6.330; −5.492), respectively; those for the BR were 8.455 (95% bootstrap confidence intervals: 8.150; 9.086) and −7.090358 (95% bootstrap confidence intervals: −7.603; −6.783), respectively. 

No significant influence of age, sex, or body-mass index, was found in any of the analyses. 

## 4. Discussion

In this manuscript, we aimed to systematically review and critically comment on studies that conducted respiratory and/or cardiac measurements using wearable devices in non-laboratory settings on patients diagnosed with PD. Our primary goal was to highlight the current opportunities and challenges in this field. Our second goal was to share our preliminary work comparing cardiorespiratory recordings by the wearable Zephyr BioPatch and the Quark-b2 stationary testing system; the inclusion of our explorative results was meant as an adjunctive contribution to the open issue of the wearables’ accuracy in the measurement of cardiorespiratory parameters. 

### 4.1. General Limitations and Comments on the Reviewed Studies 

We found very few studies suitable for review, which highlights the pioneering nature of this field. Rapid progress has been made in wearable-device technology over the last few years and researchers are only beginning to utilize this technology for non-laboratory-setting data capture. The reviewed studies represent valuable, if only preliminary, attempts to evaluate whether patients with PD have cardiorespiratory abnormalities outside of the laboratory that are similar to those found in laboratory-based testing environments [16,17,28], with the goals of predicting the occurrence of PAs using multimodal signals, including cardiac [33] and cardiorespiratory signals [29,30], and identifying the cardiac responses of patients during CBT exposure [31,32]. Overall, the reviewed studies suggest the significant potential of these new technologies to capture fine-grained, patient-specific, physiological profiles and their changes under normal environmental circumstances. However, these studies presented methodological limitations (please see Section 2.2.4, “Risk of Bias and Quality of the Reviewed Studies”), heterogeneous methodologies, and small sample sizes; in addition, confirmatory replications in independent samples are lacking and, most importantly, certain intrinsic limitations of the wearables might have undermined the reliability of the physiological recordings, as discussed below. Furthermore, the self-reporting of the experiences of PAs that were used to verify PA events (label) was highly dependent on the participants’ understanding of the nature of PA events. Thus, the ability use this input solely to develop prediction models of PA occurrence [29,30,33] potentially undermines the labeling validity. Finally, the lack of HCs in the studies assessing HR and HRV during exposure situations [31,32] prevented us from determining the extent to which the cardiac-related results were specific to PD. For these reasons, this body of research is insufficient to draw reliable conclusions about the role of cardiorespiratory function in the pathophysiology of PD and therapeutic situational exposure. Nevertheless, these studies offer important insights into the challenges related to wearable technology. These challenges are further explored in the following paragraphs, including those related to our study of the Zephyr BioPatch. 

### 4.2. Critical Wearables-Related Aspects of the Reviewed Studies and Future Research 

A critical aspect common to many of the reviewed studies was the difficulty in recording respiratory signals. The LifeShirt system provides RIP-based recordings of BR, TV, and MV, but some methodological issues can compromise the accuracy of volumetric measurements. Since RIP is an indirect approach to the estimation of volume changes, calibration procedures are needed to convert RIP-based circumferential changes in RC and AB to liters [21,36,37]. Pfaltz et al. [16,17,28] applied a qualitative diagnostic calibration with fixed-volume breathing to a 5-min quiet-sitting period. Although this is the most common calibration procedure, due to its simplicity, it was found to systematically underestimate the true values of TV and MV, with high variability in the underestimation levels among the subjects when compared to standard ergospirometry techniques for volumetric measurements [21]. The 5-minfixed-volume breathing-calibration procedure also appeared to fail in terms of accuracy when the measurement conditions or breathing patterns changed. Therefore, it only seems suitable for entire sets of breaths with unchanging or quasi-unchanging TVs [36]. Since such controlled conditions rarely occur in real-world environments, in which individuals move freely, this calibration procedure for the LifeShirt system may not be suitable for precise respiratory measurements in daily life. Furthermore, thoracoabdominal kinematics and the contribution of RC and AB to TV usually change under different postures and movements. Therefore, the performance of calibration in a single posture (e.g., only in a quiet-sitting position [16,17,28]) may undermine calibration accuracy and compromise respiratory recordings that are collected in daily life [21,47,48]. Finally, respiratory assessments based on 2-DOF systems, such as the LifeShirt system, assume that the TV is equivalent to the sum of the RC and AB volume changes. However, the accuracy of 2-DOF models in freely moving situations has also been questioned. Postural changes induce cranial–caudal displacements of the chest wall that are not covered by the RIP bands, which only capture RC and AB cross-sectional-area changes. Consequently, non-detected chest wall displacements may cause posture-related errors in volumetric estimates [36,49]. 

For all these reasons, Pfaltz et al.’s conclusion that patients with PD have very limited respiratory abnormalities in daily life, unlike the results obtained under laboratory settings [16,17,28], require replication with independent samples using approaches, which may overcome the methodological limitations induced by wearable devices. 

Several strategies have been proposed to improve the accuracy of volumetric respiratory estimates when using RIP-based, or similar, devices, such as respiratory magnetometer plethysmography (RMP). The use of reference-standard spirometry during calibration and the application of separate calibration procedures for different postures were recommended [21,36,47]. Furthermore, the application of nonlinear machine learning (NL-ML) techniques to estimate respiratory variables during calibration has also been suggested. Respiratory volumes estimated from thoracoabdominal displacements using different NL-ML models exhibited higher accuracy than linear methods in the matching of spirometry-based volumes under different conditions, such as lying, sitting, standing, and various physical exercises. Higher accuracy when using NL-ML models has also been observed during different types of breathing, including normal or constant and variable or asynchronous respiration [36,50]. The higher precision of these models probably arises from their ability to capture the complexity and variability of ventilation dynamics, which change from cycle to cycle, are associated with composite thoracoabdominal interactions, and depend on multiple variables [36,50,51,52].

Devices based on 4-DOF systems have been proposed to detect additional posture-associated chest-wall displacements for further improving the accuracy of indirect volumetric measurements. For example, RMP-based devices identify the anterior axial displacement (i.e., the variation in the distance between the xiphoid and umbilicus) and the posterior axial displacement of the spine in addition to RC and AB cross-sectional-area changes, using four coupled electromagnetic coils [51,52]. These devices provided accurate volumetric estimates for different postures: at rest, during sedentary activities, and at different levels of physical activity. However, although the results are promising and advanced versions of RMP-based wearable devices are lightweight and small [51,52], only laboratory-based studies have been performed. Future research reporting on data capture in non-laboratory settings (real-world conditions) is warranted. 

Finally, several methods have been proposed to filter measurement noise and artifacts, which may undermine the recording of physiological signals with RIP-or RMP techniques [36]. 

Given the aforementioned methodological advancements under consideration by the research community, we are confident that the accuracy of respiratory measurements can be improved. However, even recent versions of undergarment vests, smart shirts, or wearable strips and coils may still cause patient discomfort and other challenges over prolonged measurement periods in daily-life environments. Furthermore, recalibration may be necessary after the removal of these devices before their reapplication [53]. Therefore, these wearables seem to be suitable for the recording of respiratory measures during relatively limited periods in highly selected samples, whereas they may be less appropriate for prolonged use in large samples. 

To enlarge the applicability of daily-life recordings, an emerging alternative approach involves tri-axial accelerometer sensors, patched on the subject’s chest and, in some, cases on the abdomen. These sensors assess respiratory patterns by measuring the thoracoabdominal acceleration caused by respiration. Recently, wearable calibrated accelerometer sensors have appeared to be a simple, comfortable, and cost-effective solution to reliably measure respiratory rate, TV variability, and respiratory waveforms, during both sleep and wakefulness [23,54,55,56,57]. A preliminary study [23] proposed that the positioning of four accelerometers in specific positions on the subject’s thorax and abdomen may be the best combination for respiratory-waveform estimation in different postures at rest, with minimal signal-to-noise ratios. The same study also provided proof-of-concept results for the blind estimation of respiratory waveforms based on an independent-component analysis using the four accelerometers alone, suggesting that they are applicable when no reference signal is available. Since motion artifacts can undermine accelerometer-based recordings under non-resting conditions, the use of gyroscopes coupled to accelerometers may improve the reliability of respiratory measurement during a user’s physical activity [27,58]. Given that respiratory variability and waveforms are of particular interest in the pathophysiology of panic [4,59], accelerometer- and gyroscope-based respiratory measurements should be explored in patients with PD. Should reliable results be obtained, these devices may be suitable for large-scale studies over protracted recording periods, as they offer easy management and high comfort levels when worn. Longer recordings outside of the laboratory are crucial to understanding the cardiorespiratory functioning of patients with PD under multiple circumstances, making them complementary to shorter assessments under more selective conditions [16,17,28].

In line with the idea of using simple and comfortable wearables for prolonged data capture, the reviewed 3-week detection-feasibility study [29,30] used the chest-worn Zephyr BioPatch to predict oncoming PAs based on physiological data (i.e., HR, HRV, BR measured by IP, and temperature). The application prototype was also intended to notify users of their current BR and HR values while simultaneously offering breathing or relaxation exercises to pre-emptively treat imminent PA events. The prototype was built through proprietary algorithms; therefore, the methodological evaluation was severely limited. However, the authors did not provide any comments concerning the reliability of cardiorespiratory-signal recordings by the Zephyr BioPatch. 

Critical wearables-related aspects were also present in the last three reviewed studies, which provided results only on the cardiac activity recorded by different commercially available smart sports-watches (Polar RS800CX [31], the Garmin Forerunner 310XT [32], and the Garmin Vivosmart 4 [33]). 

These types of consumer-graded wearable are commonly used for non-clinical purposes, as fitness trackers during exercise-training programs. Although their application for clinical and research purposes in the medical field is increasing [60,61], the reliability of consumer wearables for fine-grained analyses on cardiac-activity recordings under different daily-life conditions is unclear. When compared to a reference-standard chest-strapped monitor during a series of sedentary and moderate physical activities in the laboratory, HR detection based on the Garmin Vivosmart wrist-worn optical HR sensors (PPG) was found to underestimate the average HR and produced some unexpected outlier readings, despite a generally acceptable accuracy [62]. Similarly, different optical devices, including the Garmin Vivosmart, provided reasonably accurate HR measurements during various physical activities in the laboratory, but presented an overall tendency to underestimate HRs in certain conditions, such as during high-intensity activities [63,64], when there was no repetitive wrist motion and when the HR signal changed quickly and at higher intensities. During activities in which the contact between the device’s sensor and skin was decreased, data capture and signal recording were negatively affected [64]. Similarly, during the in-laboratory simulation of real-world activities monitored by the Garmin Vivosmart, the individual heart-rate measurements of the older adults in one study group may have been underestimated by up to 30 beats per minute compared to an ECG-based chest strap [65]. The Garmin-based HR measurements improved when the device was in the “activity mode” setting, which probably increased the frequency with which HR was measured and instructed the device to use different algorithms, making it more suitable for detecting rapid or sudden HR changes [64]. Since the HR-measurement algorithms of smart sports-watches are generally proprietary, access to their recorded raw data might be more useful to researchers, allowing them to perform additional analyses and identify more reliable patterns of cardiac activity under different conditions. Finally, due to the generally high susceptibility of PPG-based devices to motion artifacts, several signal-processing techniques, also using simultaneously recorded accelerometer data, may remove motion-artifact effects from PPG signals and improve the detection of PPG pulses, making them suitable for performing HR estimates under various movement intensities [40,66]. 

Even when smartwatches such as the Polar RS800CX [31] and Garmin Forerunner 310XT [32] are connected to chest-strapped HR monitors, difficulties with data acquisition still arise. While chest-strapped monitors collect more accurate cardiac recordings, users’ levels of familiarity with the devices (difficulties experienced when wearing and using the recorders) and possible recorder-motion-signal loss (dropout) remain challenging issues, especially for prolonged data collection during various daily-life activities. Furthermore, the distinguishing of physical activity from the influence of emotions on cardiac activity is an important and challenging problem in the psychiatric field. To partly minimize the confounding effects of motion on cardiac signals, in one of the reviewed studies [37], only movement-free periods during different exposures were analyzed. In another study [32], only the “riding on a bus” exposure was considered, and participants were instructed to adopt a seated position while traveling. Nevertheless, the ability of these strategies to overcome motion-related limitations remains elusive. Additionally, a reliable cardiac recording during physical activity and free movement may also provide information on the pathophysiology of panic [16,28]. For these reasons, future studies should correlate cardiac recordings with movement trajectories and physical activity obtained by location tracking (GPS technology), accelerometers, and gyroscopes. 

### 4.3. Suggestions from the Pilot Comparison of Simultaneous Cardiorespiratory Recordings by the Wearable Zephyr BioPatch and the Quark-b2 Stationary Testing System 

Our explorative comparison between the Zephyr BioPatch and the reference-standard Quark-b2 stationary testing highlighted possible misestimations; therefore, the accuracy of wearables should not be assumed when they are used for clinical or research purposes. We found preliminary indications of significant bias in the two devices during at-rest recordings, especially concerning BR. Although global overlapping (i.e., a non-significant overall bias) was present between the Zephyr- and Quark-b2-based HR and BR measurements using 1-min averages, a significant variation in the bias emerged at different average values of both HR and BR. The Zephyr BioPatch overestimated both the HR and the BR at the lowest 1-min-average values and underestimated both HR and BR at the highest 1-min values. Given the wide range of values that can be encountered in HR over several minutes of recording, the HR differences we found between the two devices, although statistically significant, can be considered negligible from both clinical and research points of view [43], at least at rest. Conversely, considering the physiologically narrow range of BR values, the discrepancy we detected in the BR recording may have relevance. Finally, when we used a different duration period for the analyses (i.e., breath-by-breath instead of 1-min periods) we found an even more pronounced discrepancy between the two devices, namely a significant overall bias in both HR and BR and a larger predicted bias in both the minimum and maximum HR and BR measurements, which may have practical relevance for both cardiac and respiratory assessments. As our results were exploratory and obtained in a small sample of participants who were at rest in a sitting position, larger comparisons in different positions and at different activity levels are required. However, our findings suggest caution in the use of physiological measurements based on wearable ECG electrodes, especially concerning BR, without considering potential misestimations. Inaccurate estimates may be particularly relevant when therapeutic interventions are delivered based on these estimates, as proposed in the reviewed Zephyr BioPatch-based study [29,30]. To minimize this risk, preliminary comparisons with reference standards should be performed, at least in a subgroup of participants before they use a wearable device in an entire research sample, to evaluate possible under- or overestimations in different conditions, including different resting postures, sedentary activities, and physical activities. If the misestimations are systematic and not particularly variable in the exploratory subgroup, the analyses of the entire sample might be adjusted based on the predicted misestimation levels. Furthermore, our finding that the use of different duration periods can lead to differences in the performances of wearables suggest that the definition of a usable time frame for a specific research aim can help researchers to identify the wearable that is most suitable for providing reliable data in the timeframe of interest. Finally, achieving optimal respiration measurements with IP can greatly depend on an individual’s position because the application of certain ECG leads to patients in specific postures can capture signals better than others. Hence, the appropriate lead should be chosen to optimize the measurement or, when possible, the multiple leads should be used simultaneously to ensure the extensive capture of breathing-related signals [38]. 

Recently, we used a stepwise regression algorithm to indirectly estimate TV during exercise from wearable-device-based measurements of HR and BR [67]. Due to the preliminary nature of our analyses, we focused only on HR and BR, which are directly measured by the Zephyr BioPatch, to explore potential basal misestimations and provide “starting-point” comparisons that are easily usable without the application of inferential methods. However, the novel possibility of estimating TV from HR and BR [67], or TV variability from accelerometer-based signals [23,54,55,56,57] recorded by the Zephyr BioPatch, paves the way for further analyses to extend our comparisons to TV. 

## 5. Conclusions 

In conclusion, reliable cardiorespiratory measurements obtained by wearable devices in daily life under changing physical, psychological, or behavioral conditions may offer external and ecological validity to studies on panic pathophysiology and complement laboratory-based studies. Unfortunately, the studies reported to date were limited and several shortcomings were noted. Together with our preliminary results on the Zephyr BioPatch, these studies highlight the challenges associated with wearables-based cardiorespiratory recordings and offer an opportunity to develop solutions to improve the reliability of data collection before further work is undertaken. 

Overall, the exciting potential of these new technologies to provide valuable insights into the cardiorespiratory pathophysiology of PD outside of laboratory settings is clear. The expansion of this research is a medical need as it can contribute to the more precise phenotyping of each patient and the identification of more personalized targets for therapeutic intervention. 

## Data Availability

The data supporting the presented results of the pilot study are available on request from the corresponding author. The data are not publicly available, to protect the privacy of the participants.

## Supplementary Materials

The following supporting information can be downloaded at: https://www.mdpi.com/article/10.3390/brainsci13030502/s1, Full methodology and results of the pilot comparison of simultaneous cardiorespiratory recordings by the wearable Zephyr BioPatch and the Quark-b2 stationary testing system: Pilot comparison of simultaneous cardiorespiratory recordings by the wearable Zephyr BioPatch and the Quark-b2 stationary testing system.

## Abbreviations

AB	Abdomen
B&A	Bland–Altman-plot analysis
BR	Breathing rate
CBT	Cognitive-behavioral therapy
DCS	D-cycloserine
DOF	Degrees of freedom
ECG	Electrocardiogram
HCs	Healthy controls
HR	Heart rate
HRV	Heart-rate variability
IP	Impedance pneumography
ML	Machine learning
MV	Minute ventilation
NL	Nonlinear
PA(s)	Panic attack(s)
PD	Panic Disorder
PPG	Photoplethysmography
RC	Rib cage
RMP	Respiratory magnetometer plethysmography
RIP	Respiratory inductive plethysmography
TV	Tidal volume
