# Supplementary material for: Cardiorespiratory Assessments in Panic Disorder Facilitated by Wearable Devices: A Systematic Review and Brief Comparison of the Wearable Zephyr BioPatch with the Quark-b2 Stationary Testing System
Source: Brain Sci. 2023 Mar 16;13(3):502. doi: 10.3390/brainsci13030502 (PMC10046237; doi:10.3390/brainsci13030502)
Supplement: Supplementary file 1 [file brainsci-13-00502-s001.zip › brainsci-2216896-supplementary.pdf]

Supplementary Materials

Pilot comparison of simultaneous cardiorespiratory recordings by the wearable Zephyr BioPatch and the Quark-b2 stationary testing system

Materials and Methods

Study Design

This is a pilot cross-sectional study to evaluate the accuracy of heart rate (HR) and breathing rate (BR) recordings by the wearable Zephyr BioPatch compared to the Quark-b2 stationary testing system, during a simultaneous recording. This study is a part of a larger ongoing longitudinal observational study carried out at Villa San Benedetto Menni Hospital, Albese con Cassano, Como, Italy, entitled "Effects of treatments on quality of life of patients with psychiatric disorders with or without medical comorbidity." The entire observational study encompassed the possibility of recruiting volunteers from non-clinical populations to obtain comparison data. This entire observational study adheres to the principles of the Helsinki Declaration and has been approved by the Ethical Committee of the Local Health Authority of the Province of Como, Italy (Protocol Number: 0021453 first approval: 1 July 2010, with subsequent four-year renewals until October 2026).

Participants

10 participants (5 males and 5 females) were recruited among staff form the Villa San Benedetto Menni Hospital and included in this pilot study. Outside of obtaining written informed consent from the participants, no inclusion or exclusion criteria were applied. The sample's average age was 48.6±13.1 years (range: 28-65) and the average body-mass index (BMI) was 22.9±3.2 (range: 18.9-30.1). All participants were Caucasians and 2 (20%) were smokers.

Instruments

The Quark-b2 stationary system

The Quark-b2 stationary testing system (Cosmed, Italy) (Figure S2) assesses respiration physiology by monitoring respiratory functioning and pulmonary gas exchange on a breath-by-breath basis. It also assesses cardiac activity using a 12 lead ECG monitoring to provide hearth rate (HR) and heart rate variability (HRV) estimation. The Quark-b2 system reports BR

and HR every breath, with precision to the second. The system consists of a mobile unit containing the principal components, which are connected to a computer to allow continuous breath-by-breath recording of respiratory parameters. The principal components are 1) a digital infrared-light turbine that detects respiration airflows, 2) rapid-response O2 and CO2 analyzers, 3) electronic sensors measuring barometric pressure, ambient temperature, and humidity, 4) a humidity absorber, and 5) an ECG module. An open, light face mask connects the subject to the respiratory testing system; an antiviral/antibacterial filter is placed in between the turbine and the mask. Before each test, the turbine and the analyzers were calibrated in order to maintain optimal technical characteristics of the apparatus.
The Quark-b2 is a validated instrument widely used in sports medicine and respiration physiology studies, in accordance with the recommendations of the American Thoracic Society and the European Respiratory Society [1].

The Zephyr BioPatch

A detailed description of the Zephyr BioPatch (Zephyr BioPatch, Medtronic, Inc.) (Figures S1 and S2) is provided in the article (3. Section II: Pilot comparison of simultaneous cardiorespiratory recordings by the wearable Zephyr BioPatch and the Quark-b2 stationary testing system. 3.1 Materials and Methods). It is a chest-worn patch-style device that has to be placed in the user's epigastric quadrant of the subxiphoid region. It continuously records multiple physiological signals and provides the related outputs, including HR and breathing rate (BR) per minute, as calculated by internal proprietary algorithms. Outputs are reported every second, with precision to the millisecond.

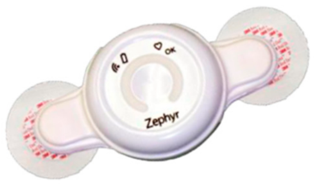

Figure S1. The Zephyr BioPatch

Procedure

In each participant, we simultaneously recorded respiratory and cardiac signals by the Zephyr BioPatch and Quark-b2 system. The cardiorespiratory recordings were carried out by some of

the Authors (A.A.; D.C.; S.D.; M.G.; W.S.) that have been trained in the use of the Quark-b2 system and Zephyr BioPatch. A standardized procedure was used throughout to minimize any confounding influences. Recordings were taken over 20 min and captured at rest and in a sitting position in a quiet room.

After the placement of the mask, antiviral/antibacterial filter, and ECG leads, the subject was given 5 min to rest and familiarize her/himself with the study apparatus before the recording began. The following leads, sufficient to the aim of the study, were placed: the Right Upper Limb lead below the right clavicle and near the right shoulder; the Left Upper Limb lead below the left clavicle and near the left shoulder; the Left Lower Limb lead above the iliac crest at the interception with the midclavicular line; and the Neutral Limb lead in the same position on the right side. During the entire session, the subjects were instructed to remain seated silently and that they could end the session at any time by giving the examiner a hand signal. Throughout the procedure, the examiners kept track of the continuous breath by breath Quark-b2 recording on a computer screen. After each recording, the face masks were sanitized.

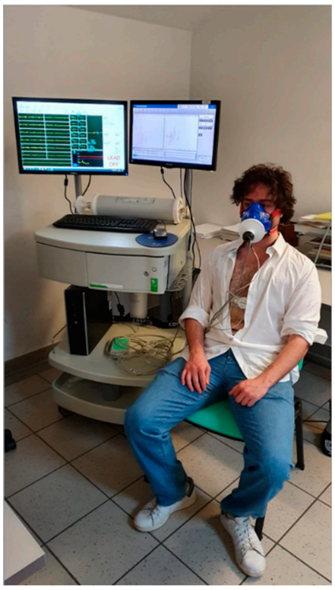

Figure S2. Experimental setup.

A participant is connected to the Quark-b2 system and wears the Zephyr BioPatch

Calculation of BR and HR from signals of the Quark-b2 system and the Zephyr BioPatch

The Quark-b2 system reports BR and HR every breath, with precision to the second, whereas the Zephyr BioPatch reports them every second, with precision to the millisecond; therefore, the BR and HR parameters had to be synched and recalculated within identical periods to analyze the agreement between the two devices.

To synch the measurements, the timestamp provided by the Zephyr BioPatch was rounded to seconds to match the Quark-b2 timestamp resolution (i.e., in case the milliseconds of the timestamp were >500, the Zephyr BioPatch timestamp was rounded to the next second; otherwise, milliseconds were trunked). Since for technical reasons the start and end of recordings with the 2 devices did not happen exactly at the same time, only the parts of the Zephyr BioPatch recordings that overlapped those of the Quark b2 were retained.

To recalculate the measurements within identical periods, at first BR and HR from both Quark-b2 and Zephyr BioPatch were recalculated in epochs of 1-min length. The recording time was split in epochs of 1-min, and we identified which BR and HR measurements, both from Quark-b2 and Zephyr BioPatch, were related to each epoch. In the Quark-b2 recordings, in which the measurements are provided per each breath, some breaths may have been only partially included in a certain epoch, as the beginning or end of the breath may have occurred before or after the beginning or end of a certain epoch. Thus, only the measurements related to those breaths whose beginning and end occurred within the epoch of interest were considered as part of that epoch. The Zephyr BioPatch BR and HR for each epoch were calculated as the simple average of all measurements in that epoch. Measurements whose confidence parameter (range 0-100) was indicated as less than 50 were excluded from the analysis. In case an epoch had less than 50% Zephyr BioPatch measurements with confidence ≥ 50, that entirely epoch was excluded from the analysis. Instead, the Quark-b2 BR and HR for each 1-min epoch was calculated as the average of all measurements in that epoch, weighted by the length of each breath. The use of a weighted average was necessary because the measurements performed by Quark-b2 were not provided in fixed-length time windows.

Finally, the breaths detected by Quark-b2 were considered as a further time unit used in the analysis, and the BR and HR measurements from Zephyr BioPatch have been recalculated to match every breath detected by Quark b2. For this aim, we considered the native breath-by-breath Quark-b2 measurements of BR and HR, and we identified the BR and HR Zephyr BioPatch measurements that were related to each breath identified by Quark b2. Confidence parameters (range 0-100) was applied to the Zephyr BioPatch measurements, as described above.

Statistical analysis

To evaluate the level of agreement between the measurements provided by the two devices, we used the Bland-Altman plot analysis [2,3], which allows visual and quantitative comparison of

two measurements techniques by plotting the measurement differences of the two techniques against the average of the measurements obtained with the two techniques.

Specifically, in the Bland-Altman plot analysis we considered the bias, the lower-bound limit of agreement, the upper-bound limit of agreement, and their respective 95% confidence intervals, to evaluate the observed agreement between the measurements of BR and HR provided by the Zephyr BioPatch and the Quark-b2 system.

Given that multiple measurement epochs from each subject were used in the analysis, a multi-level (mixed-model) limit of agreement analysis have been performed. According to Parker and coauthors [4], we used the *loa_mixed* function available in the *SimplyAgree* R library, version 0.0.3 (https://CRAN.R-project.org/package=SimplyAgree) [5]. The use of the *loa_mixed* function has the additional advantage that confidence intervals of bias and limit of agreement are calculated non-parametrically by a bootstrap resampling procedure (1000 resamples), which removes the usual Bland-Altman plot assumption of a normality distribution of the difference between measurements of the two methods.

Finally, to investigate if the level of bias between the two devices resulted different at different levels of measurement, or at different age or BMI levels, or in different sex, we performed a linear mixed-model (regression) analysis with the difference between the two methods as dependent variable and the average measurement between the two method, age, sex, and BMI as fixed effect. A random effect of the intercept with subjects as grouping factor was also added in the model. A significance in the regression model coefficient indicates that the bias level significantly changes at different values of the fixed effects. In addition, in case of a significance in the regression model coefficient of the average measurement between the two methods, the predicted bias at the minimum and maximum observed average measurement values was investigated to evidence a possible inversion of sign in the bias along the range of measurement values.

The abovementioned analyses were applied to both the measurements considering epoch of 1-min and the Quark-b2 breath-by-breath measurements.

We set the level of significance at the conventional 0.05 and performed the analyses by the R programming language version 3.6.3 (*R Core Team, R: A Language and Environment for Statistical Computing, R Foundation for Statistical Computing, Vienna, Austria, 2020*)

Results

In the entire sample, 2496 breath-by-breath measurements were obtained by the Quark-b2 system and 11938 one-second measurements were obtained by the Zephyr BioPatch, with a

total of 198 min and 58 s of simultaneous recordings with the two devices. The mean recording time for each participant was 19 min and 51 s (standard deviation (SD) = 1 min and 51 s), with a mean of 249.6 breaths recorded by Quark-b2 for each participant (SD= 102.88) and a mean of 1193.8 one-second recordings by Zephyr for each participant (SD= 110.9).

Among the Zephyr BioPatch recordings, 6.36% (n=759) of the one-second HR recordings and 8.15% (n=973) of the one-second BR recordings had a confidence score <50 and were excluded from the analyses, whereas the remaining 11179 HR and 10965 BR recordings were included. After matching signals of the two device, 188 complete 1-min epochs resulted. Twelve of these (6.38%) were removed for the analysis of HR and 13 (6.91%) were removed from the analysis of BR because less than 50% Zephyr BioPatch measurements had a confidence level ≥ 50. Thus, 176 1-min epochs were used in the analysis of HR and 175 1-min epochs were used in the analysis of BR.

Finally, of the 2496 breaths detected by Quark b2, 153 (6.13%) were removed for the analysis of HR and 197 (7.89 %) were removed for the analysis of BR because less than 50 Zephyr measurements related to those breaths had a confidence level ≥ 50. Therefore, 2343 breaths were used in the analysis of HR and 2299 breaths were used in the analysis of BR.

Heart Rate

In the **Bland-Altman plot analysis of the 1-min average HR (Figure S3)**, the overall bias resulted non-significantly different than zero (bias= 0.078; 95% bootstrap confidence intervals: -0.087; 0.243). However, the mixed-model analysis revealed a statistically significant variation of the bias at different average value of HR ($B= 0.539$, $p < 0.001$). The predicted bias at the minimum observed 1-min average HR value (60.342) resulted -1.046, indicating an expected overestimation of the Zephyr BioPatch compared to Quark b2. Instead, the predicted bias at the maximum observed 1-min average HR value (101.81) resulted 1.912, indicating an expected overestimation of the Zephyr BioPatch compared to Quark b2. No statistically significant variations of the bias at different age ($B = 0.043$), sex ($B = -0.169$), or BMI ($B = 0.100$) were found.

The upper- and lower-bound limit of agreement resulted respectively 2.044 (95% bootstrap confidence intervals: 1.506; 2.413) and -1.812 (95% bootstrap confidence intervals: -2.109; -1.373).

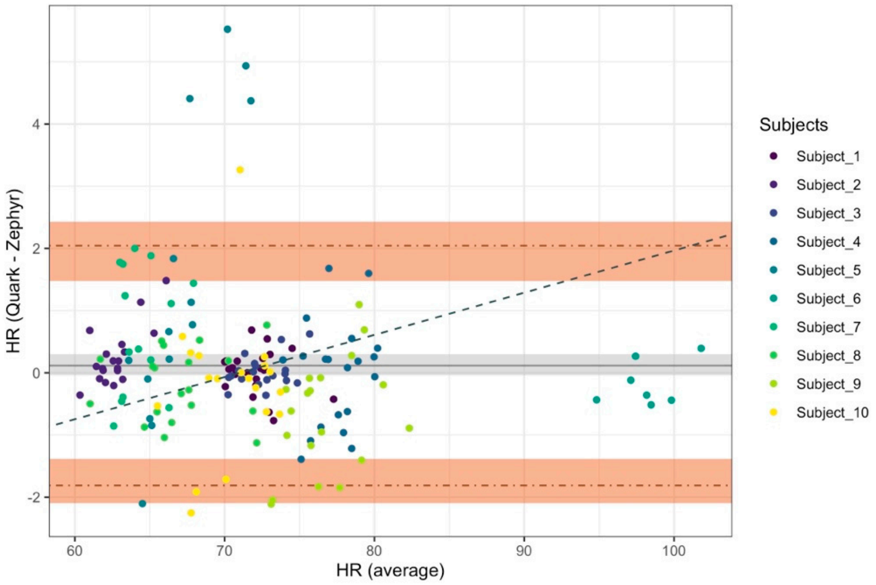

Figure S3. Bland-Altman plot of the 1-min average HR

In the **Bland-Altman plot analyses at the level of the breath-by-breath Quark-b2 measurements (Figure S4)**, the overall bias resulted statistically significant different than zero (bias= 0.212; 95% bootstrap confidence intervals: 0.079; 0.390), indicating an average overestimation of Zephyr BioPatch compared to Quark b2. Moreover, the mixed-model analysis revealed a statistically significant variation of the bias at different average value of HR (B = 0.4239, p < 0.001). The predicted bias at the minimum observed breath-by-breath Quark-b2 HR measurement (56) resulted -5.155, indicating an expected overestimation of the Zephyr BioPatch compared to Quark b2. Instead, the predicted bias at the maximum observed breath-by-breath Quark-b2 HR measurement (106) resulted 16.65, indicating an expected underestimation of the Zephyr BioPatch compared to Quark b2. No statistically significant variations of the bias at different age (B = 0.043), sex (B = -0.169), or BMI (B = 0.100) were found.

The upper- and lower-bound limit of agreement resulted respectively 6.352 (95% bootstrap confidence intervals: 5.886; 6.897) and -5.9287675 (95% bootstrap confidence intervals: -6.330; -5.492).

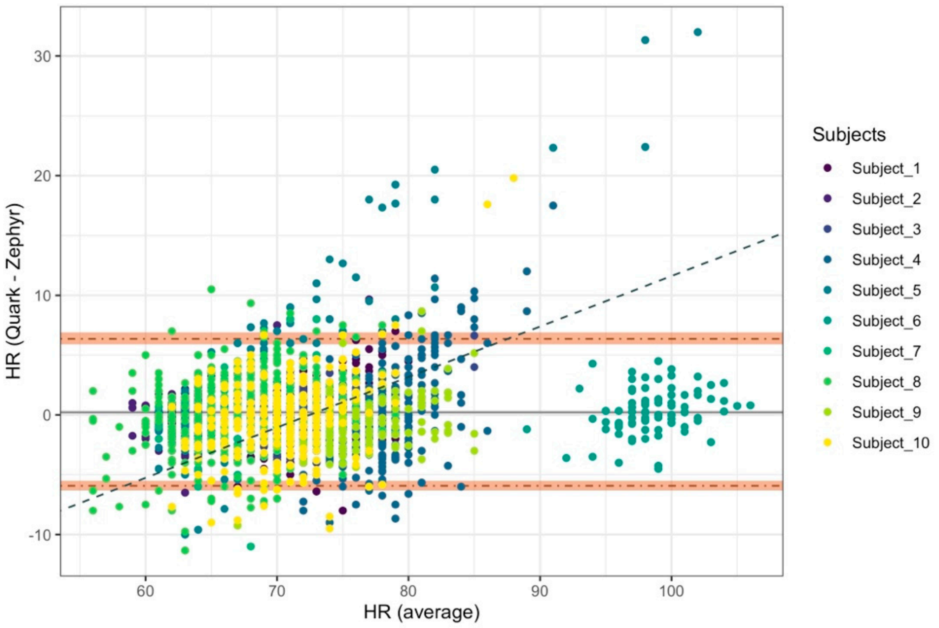

Figure S4. Bland-Altman plot at the level of the breath-by-breath Quark-b2 measurements of HR

Breathing rate

In the **Bland-Altman plot analyses of the 1-min average BR (Figure S5)**, the overall bias resulted non-significantly different than zero (bias = 0.098; 95% bootstrap confidence intervals: -0.177; 0.396). However, the mixed-model analysis revealed a statistically significant variation of the bias at different average value of BR (B = 0.10283, $p < 0.001$). The predicted bias at the minimum observed 1-min average BR value (5.431) resulted -5.1, indicating an expected overestimation of the Zephyr BioPatch compared to Quark b2. Instead, the predicted bias at the maximum observed 1-min average BR value (24.45) resulted 4.123, indicating an expected underestimation of the Zephyr BioPatch compared to Quark b2. No statistically significant variations of the bias at different age (B = -0.006), sex (B = 0.111), or BMI (B = 0.142) were found.

The upper- and lower-bound limit of agreement resulted respectively 3.705 (95% bootstrap confidence intervals: 2.617; 4.584) and -3.51 (95% bootstrap confidence intervals: -4.145; -2.661).

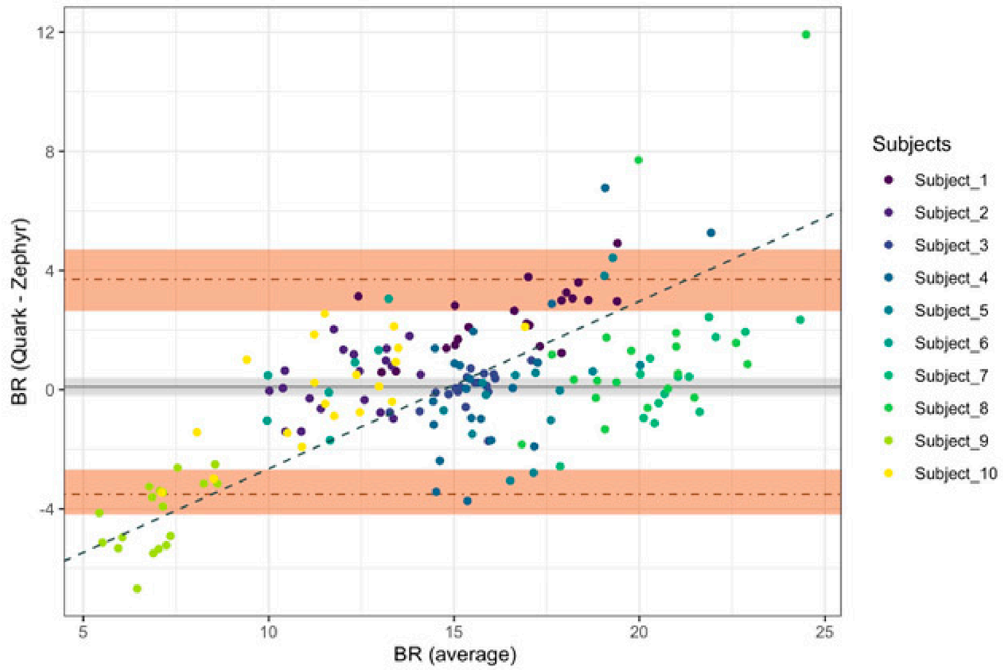

Figure S5. Bland-Altman plot of the 1-min average BR

In the **Bland-Altman plot analyses at the level of the breath-by-breath Quark-b2 measurements (Figure S6)**, the overall bias resulted statistically significant (bias = 0.682; 95% bootstrap confidence intervals: 0.538; 0.897), indicating an average overestimation of Zephyr BioPatch compared to the Quark b2. Moreover, the mixed-model analysis revealed a statistically significant variation of the bias at different average value of BR (B = 0.955, p < 0.001). The predicted bias at the minimum observed breath-by-breath Quark-b2 BR measurement (3.23) resulted -11.844, indicating an expected overestimation of the Zephyr compared to Quark b2. Instead, the predicted bias at the maximum breath-by-breath Quark-b2 BR measurement (48.39) resulted 31.024, indicating an expected underestimation of the Zephyr compared to Quark b2. No statistically significant variations of the bias at different age (B = -0.050), sex (B = 0.313), and BMI (B = 0.328) were found.

The upper- and lower-bound limit of agreement resulted respectively 8.455 (95% bootstrap confidence intervals: 8.150; 9.086) and -7.090358 (95% bootstrap confidence intervals: -7.603; -6.783).

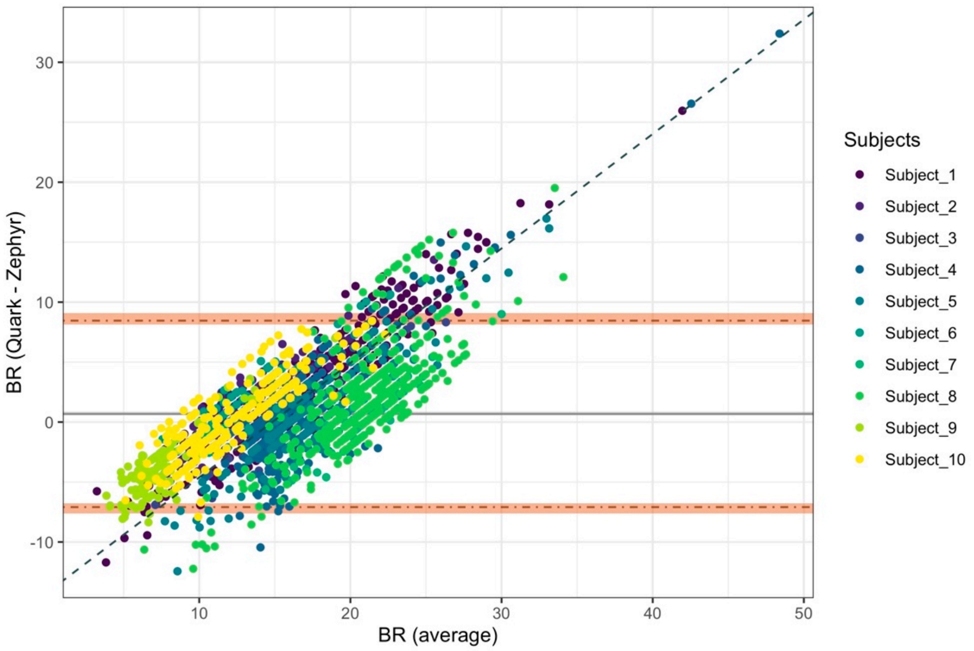

Figure S6. Bland-Altman plot at the level of the breath-by-breath Quark-b2 measurements of BR

References

1. Caldirola, D.; Bellodi, L.; Caumo, A.; Migliarese, G.; Perna, G. Approximate Entropy of Respiratory Patterns in Panic Disorder. *Am. J. Psychiatry* **2004**, *161*, 79–87, doi:10.1176/APPI.AJP.161.1.79.
2. Giavarina, D. Understanding Bland Altman Analysis. *Biochem. medica* **2015**, *25*, 141–151, doi:10.11613/BM.2015.015.
3. Bland, M.J.; Altman, D.G. Statistical Methods for Assessing Agreement between Two Methods of Clinical Measurement. *Lancet (London, England)* **1986**, *1*, 307–310, doi:10.1016/S0140-6736(86)90837-8.
4. Parker, R.A.; Weir, C.J.; Rubio, N.; Rabinovich, R.; Pinnock, H.; Hanley, J.; McCloughan, L.; Drost, E.M.; Mantoani, L.C.; MacNee, W.; et al. Application of Mixed Effects Limits of Agreement in the Presence of Multiple Sources of Variability: Exemplar from the Comparison of Several Devices to Measure Respiratory Rate in COPD Patients. *PLoS One* **2016**, *11*, doi:10.1371/JOURNAL.PONE.0168321.
5. Caldwell, A.R. SimplyAgree: An R Package and Jamovi Module for Simplifying Agreement and Reliability Analyses. *J. Open Source Softw.* **2022**, *7*, 4148, doi:10.21105/JOSS.04148.
